# Health Belief Model and HIV/AIDS among high school female students in Yazd, Iran

**Published:** 2010

**Authors:** Mohammad Hossein Baghianimoghaddam, Hossein Forghani, Razie Zolghadr, Zohre Rahaii, Parisa Khani

**Affiliations:** a Faculty of Health, Shahid Sadoughi University of Medical Sciences, Yazd, Iran; b Research and Clinical Center for Infertility, Shahid Sadoughi University of Medical Sciences, Yazd, Iran

Acquired immune deficiency syndrome (AIDS) is a disease of the human immune system. It is caused by the human immunodeficiency virus (HIV) which can be transmitted through direct contact of a mucous membrane or the bloodstream with a bodily fluid containing HIV.^1^–^4^ It is such a strange and frightening disease that sometimes people stop seeing it as a disease and wrap it in different layers of mystery.^5^ According to the latest figures published in the UN-AIDS/WHO, AIDS Epidemic Update 2009, an estimated 33.4 million people were living with HIV in 2008.^6^ An important factor in the spread of HIV/AIDS is believed to be poor knowledge about how it is spread and how it can be prevented. The utility of the Health Belief Model (HBM) continues to be suggested in identifying preventive behaviors. This study is a cross-sectional study in which 180 female students from three high schools in Yazd, Iran, completed a specially designed questionnaire, based on HBM in spring 2009. Data showed that the mean score of perceived susceptibility was 21.19 (out of 32) and it was 12.47 (out of 24) for perceived severity. Also the mean scores of perceived benefits and barriers were 9.05 and 9.45 (out of 12 and 16, respectively). The respondents acquired 46.61% of total knowledge score, 66.21% of perceived susceptibility, 51.95% of perceived severity, 75.41% of perceived benefits and 59.06% of perceived barriers. A positive association found between knowledge and perceived susceptibility. Therefore, according to the results, the low level of perceived susceptibility to and severity of HIV/AIDS among high school female students may not prevent them from trying risky behaviors. The results of this study revealed that participants’ mean scores of perceived severity of HIV/AIDS was near average. These results also reveal that the participants do not believe that they are in high risk of HIV/AIDS. Having such a belief, students do not try to prevent HIV/AIDS. In a factor analysis, principal components of the HBM constructs indicated that perceived susceptibility was multidimensional. So, sporadic condom users perceived themselves and their partners as at the highest risk of AIDS and other sexually transmitted diseases.^7^ In that study, prisoners decreased their HIV high-risk behaviors (e.g., used clean syringes) when they believed in the effectiveness of strategies designed to reduce the risk or in seriousness of their impact of the health condition. This does not mean that the other two components of the HBM are not effective in explaining health related behavior.

There was a significant difference between mean scores of knowledge and perceived susceptibility and educational field of participants (p < 0.001).The negative significant correlation between perceived severity with knowledge and perceived susceptibility indicates that the students who have not perceived susceptibility to and severity of a health problem like HIV/AIDS may ignore preventing behaviors. Moreover, the Health Belief Model can be used as a conceptual framework in interventional programs for HIV/AIDS.
